# An Italian multicentre distributed data research network to study the use, effectiveness, and safety of immunosuppressive drugs in transplant patients: Framework and perspectives of the CESIT project

**DOI:** 10.3389/fphar.2022.959267

**Published:** 2022-09-15

**Authors:** Valeria Belleudi, Alessandro C. Rosa, Marco Finocchietti, Francesca R. Poggi, Maria Lucia Marino, Marco Massari, Stefania Spila Alegiani, Lucia Masiero, Andrea Ricci, Gaia Bedeschi, Francesca Puoti, Massimo Cardillo, Silvia Pierobon, Maurizio Nordio, Eliana Ferroni, Martina Zanforlini, Giuseppe Piccolo, Olivia Leone, Stefano Ledda, Paolo Carta, Donatella Garau, Ersilia Lucenteforte, Marina Davoli, Antonio Addis, CESIT Study Group, Antonio Addis

**Affiliations:** ^1^ Department of Epidemiology, Lazio Regional Health Service, Rome, Italy; ^2^ National Center for Drug Research and Evaluation, Istituto Superiore Di Sanità, Rome, Italy; ^3^ Italian National Transplant Center—Istituto Superiore Di Sanità, Rome, Italy; ^4^ Azienda Zero of the Veneto Region, Padua, Italy; ^5^ ARIA, S.p.a., Milan, Italy; ^6^ Regional Transplant Coordination, Milan, Italy; ^7^ Directorate General for Health, Milan, Italy; ^8^ General Directorate for Health, Sardinia Region, Italy; ^9^ Department of Clinical and Experimental Medicine, University of Pisa, Pisa, Italy; ^10^ CESIT Study Group is a collaborative author

**Keywords:** immunosuppressive treatment, transplant, research network, distributed analysis, pharmacoepidemiology, pharmaco-utilization, multidisciplinary approach

## Abstract

The goal of post-transplant immunosuppressive drug therapy is to prevent organ rejection while minimizing drug toxicities. In clinical practice, a multidrug approach is commonly used and involves drugs with different mechanisms of action, including calcineurin inhibitors (CNI) (tacrolimus or cyclosporine), antimetabolite (antimet) (mycophenolate or azathioprine), inhibitors of mechanistic target of rapamycin (mTOR) (sirolimus or everolimus), and/or steroids. Although evidence based on several randomized clinical trials is available, the optimal immunosuppressive therapy has not been established and may vary among organ transplant settings. To improve the knowledge on this topic, a multiregional research network to Compare the Effectiveness and Safety of Immunosuppressive drugs in Transplant patients (CESIT) has been created with the financial support of the Italian Medicines Agency. In this article, we describe the development of this network, the framework that was designed to perform observational studies, and we also give an overview of the preliminary results that we have obtained. A multi-database transplant cohort was enrolled using a common data model based on healthcare claims data of four Italian regions (Lombardy, Veneto, Lazio, and Sardinia). Analytical datasets were created using an open-source tool for distributed analysis. To link the National Transplant Information System to the regional transplant cohorts, a semi-deterministic record linkage procedure was performed. Overall, 6,914 transplant patients from 2009–19 were identified: 4,029 (58.3%) for kidney, 2,219 (32.1%) for liver, 434 (6.3%) for heart, and 215 (3.1%) for lung. As expected, demographic and clinical characteristics showed considerable variability among organ settings. Although the triple therapy in terms of CNI + antimet/mTOR + steroids was widely dispensed for all settings (63.7% for kidney, 33.5% for liver, 53.3% for heart, and 63.7% for lung), differences in the active agents involved were detected. The CESIT network represents a great opportunity to study several aspects related to the use, safety, and effectiveness of post-transplant maintenance immunosuppressive therapy in real practice.

## Introduction

In the solid organ transplant setting, different immunosuppressive drug regimens are administrated in the maintenance phase to prevent organ rejection. Although the use of these drugs is essential for the survival of the patient, they can be responsible for several types of adverse outcomes, in terms of both disease onset (e.g., diabetes or hypertension) and possible iatrogenic disorders (e.g., cancer or infections) (Kasiske et al., 2010).

The current standard immunosuppressive therapy in most kidney transplant protocols recommends a maintenance phase with calcineurin inhibitor (CNI) [i.e., tacrolimus (TAC) or cyclosporine (CsA)], in combination with an antiproliferative agent (antimet) [i.e., mycophenolate (MMF) or azathioprine (AZA)] or an inhibitor of mechanistic target of rapamycin (mTOR) [i.e., sirolimus (SIR) or everolimus (EVE)], with or without corticosteroids (Kasiske et al., 2010; Heemann et al., 2011). In the case of liver transplant, there is no standard-of-care designation for immunosuppression choice, although current practice includes CNI-based regimens (McGuire et al., 2009; AISF). For heart, lung, and other transplants, the choice of immunosuppressive therapy is in charge of a single center. Direct comparative trials have only been conducted in the case of CNI and in the kidney setting. A meta-analysis of such trials showed that TAC is superior to CsA in preventing acute rejection, even though this could be at the expense of increased risk of diabetes (Webster et al., 2005). The same results were also confirmed in patients with liver transplant (Muduma et al., 2016).

The recent availability of generic immunosuppressive drug therapies on the market has raised discussion among the scientific and regulatory community at national and international level, regarding switching or replacement among different brand names of the same substance (Cattaneo et al., 2005; ESOT Advisory Committee on Generic Substitution, 2011; Molnar et al., 2015; Kahn et al., 2020).

The development of the extended-release formulation for TAC has drawn the attention of researchers to the need to compare the two formulations (i.e., prolonged versus immediate) in clinical practice. In fact, although several randomized clinical trials (RCTs) have demonstrated equivalence in terms of efficacy and safety between the two formulations, the exclusion of patients with high immunological risk from trials raises doubts about the generalizability of these results (Vadcharavivad et al., 2019).

Evidence of comparative efficacy and safety of immunosuppressive drugs is limited. The main meta-analyses include small RCTs with a short follow-up that fails to evaluate possible differences between subgroups. In addition, they do not take into account the different formulations of immunosuppressive drugs and their combinations (Webster et al., 2005; Haasova et al., 2016; Muduma et al., 2016; Azarfar et al., 2018; Hahn et al., 2019). Data on generic use in solid transplantation are even more scarce (Mansukhani and Conway, 2015).

It is known that patients enrolled in RCTs are highly selected: the elderly are underrepresented and clinical practice in pediatric patients is derived from adult studies. Furthermore, severe and robust outcomes (e.g., mortality, rejection, infection, and cancer) are more difficult to detect, requiring a very long follow-up.

The difficulty of investigating these results in clinical trials makes it necessary to conduct comparative observational studies on the effectiveness and safety of immunosuppressive drugs to have longer and stronger end-point follow-up. In addition, this kind of study may be useful to compare different post-transplant immunosuppressive strategies used in clinical practice and investigate their determinants.

Moreover, observational studies may be useful to analyze specific issues, such as adherence, switching, and factors associated with generic drug uptake.

To improve our knowledge of maintenance immunosuppressive therapies prescribed after solid organ transplant, a multiregional research network was developed with the financial support of the Italian Medicines Agency (AIFA). The aim was to study drug use and compare the effectiveness and safety of immunosuppressive drugs in transplant patients (CESIT). In this paper, we describe the development of the CESIT network, the main aspects of the framework designed to perform observational research studies within the network, and we will also give an overview of the principal characteristics, including drug regimen, by type of organ transplant.

## Methods and materials

### Participants and organizations

**FIGURE 1:**
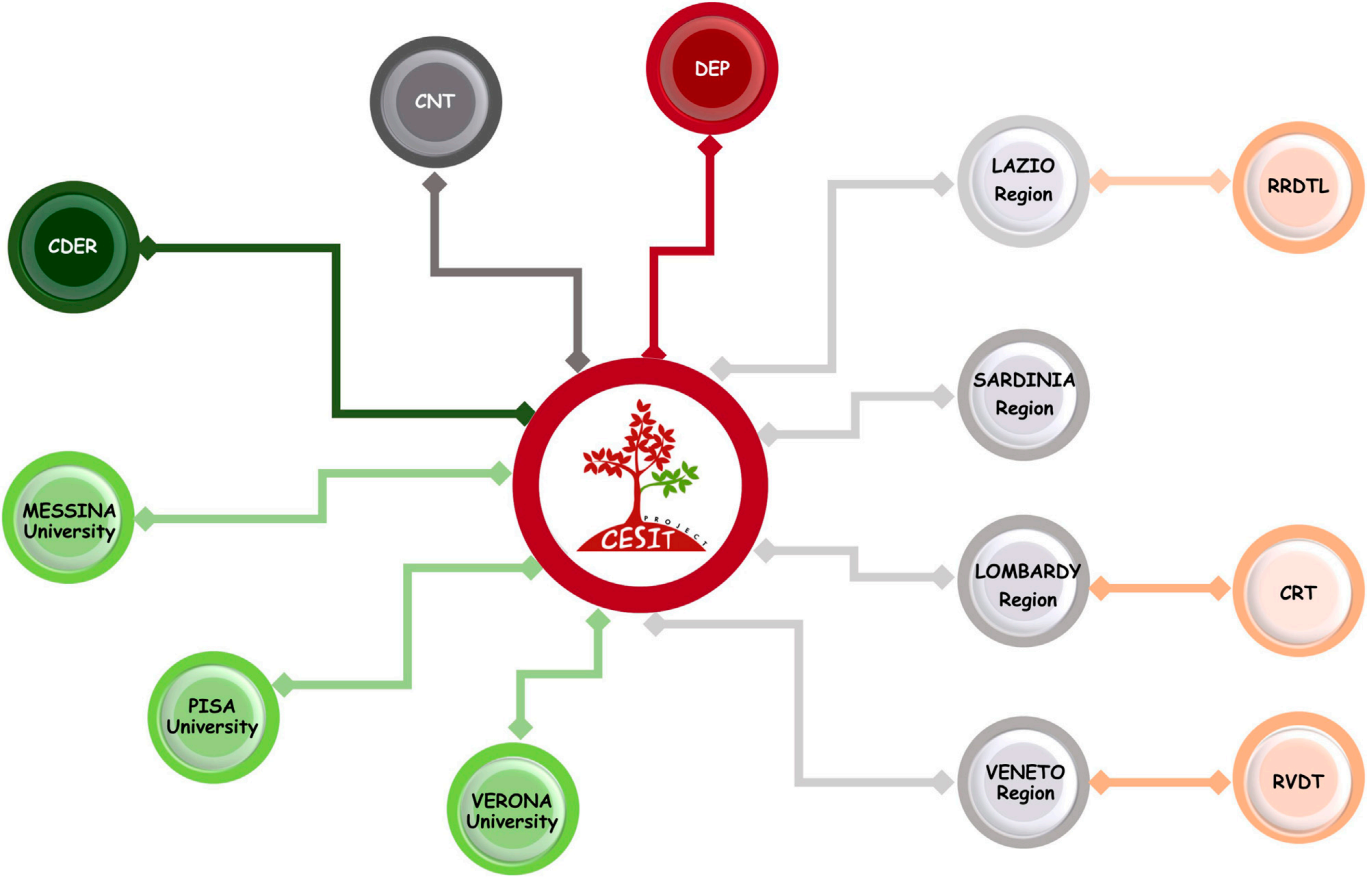
CESIT research network: participants and organization. Note. DEP: Department of Epidemiology, Lazio region; RRDTL: Lazio regional dialysis and transplant register; RVDT: Veneto regional dialysis and transplant register; CRT: Lombardy regional transplant center; CNT: National Transplant Centre; CDER: National Centre for Drug Research and Evaluation.

The CESIT research network is composed of several scientific and institutional organizations under the coordination of the Department of Epidemiology of the Lazio region, DEP, which has specific skills in pharmacoepidemiology, data analysis, and statistical models. Specifically, the network consists of four Italian regions (Lombardy, Veneto, Lazio, and Sardinia), accounting for an overall population of ∼22.5 million inhabitants. Regions participate in the project through the sharing of analytical dataset extracted by regional health claims. In particular for Veneto and Lazio regions, data retrieved in dialysis and kidney transplant registries are also available. In addition, two departments of Italian National Institute of Health are involved in the network: the National Transplant Centre (CNT) and the National Centre for Drug Research and Evaluation (CDER). The CNT, which was established by Law 91/99 as the national competent authority for organ donation and transplantation activities (Italian National Transplant Center, 2022), contributes to the project through specific know-how regarding solid transplant area and sharing national data on solid organ transplants, collected prospectively, and then makes data available through an ad hoc data flow. The CDER collaborates to the project by specific pharmacoepidemiologic expertise and providing an R-based open-source tool, “TheShinISS,” which was developed for distributed data analysis (Massari et al., 2020; Trifirò et al., 2021). Finally, several experts in transplantation, pharmacoepidemiology, and pharmacovigilance research from public and academic institutions have been involved in the network (Figure 1).

### Project’s objectives

The project’s aims are as follows:

• To describe dispensation patterns of immunosuppressive drug regimens in different transplant organ settings (i.e., kidney, liver, heart, and lung), considering several special populations (i.e., elderly and pediatrics) and identifying specific factors, determinants of use, which are associated with immunosuppressive drug regimens in the maintenance phase.
• To compare the risk-benefit profile of different immunosuppressive therapeutic regimens, including generics and originators, in the post-transplant maintenance phase.
• To evaluate data validity and results generalizability through the National Transplant Information System (SIT). Analyze the concordance between dispensation pattern registered by SIT and regional claims data.

### Data sources

The Italian National Health Service (NHS) is a system of structures and services whose purpose is to guarantee universal access to health services to all citizens, on equal terms. It covers, totally or partially (by a system of health care cost-sharing for patients called “ticket”), a wide range of pharmaceuticals and diagnostic services that are essential for health. The NHS promotes, through the local health authorities, efficient and effective management to ensure uniformity of service delivery across the country. In this context, Italian healthcare administrative data have a high level of coverage because data collection on a regional basis is mandated by national law across the whole country to track healthcare service utilization and to monitor uniformity of service delivery across the country. Most of these data sources have been extensively used to study several aspects of healthcare, including drug utilization, safety, comparative effectiveness, cost, and cost-effectiveness (Trifirò et al., 2019).

**FIGURE 2:**
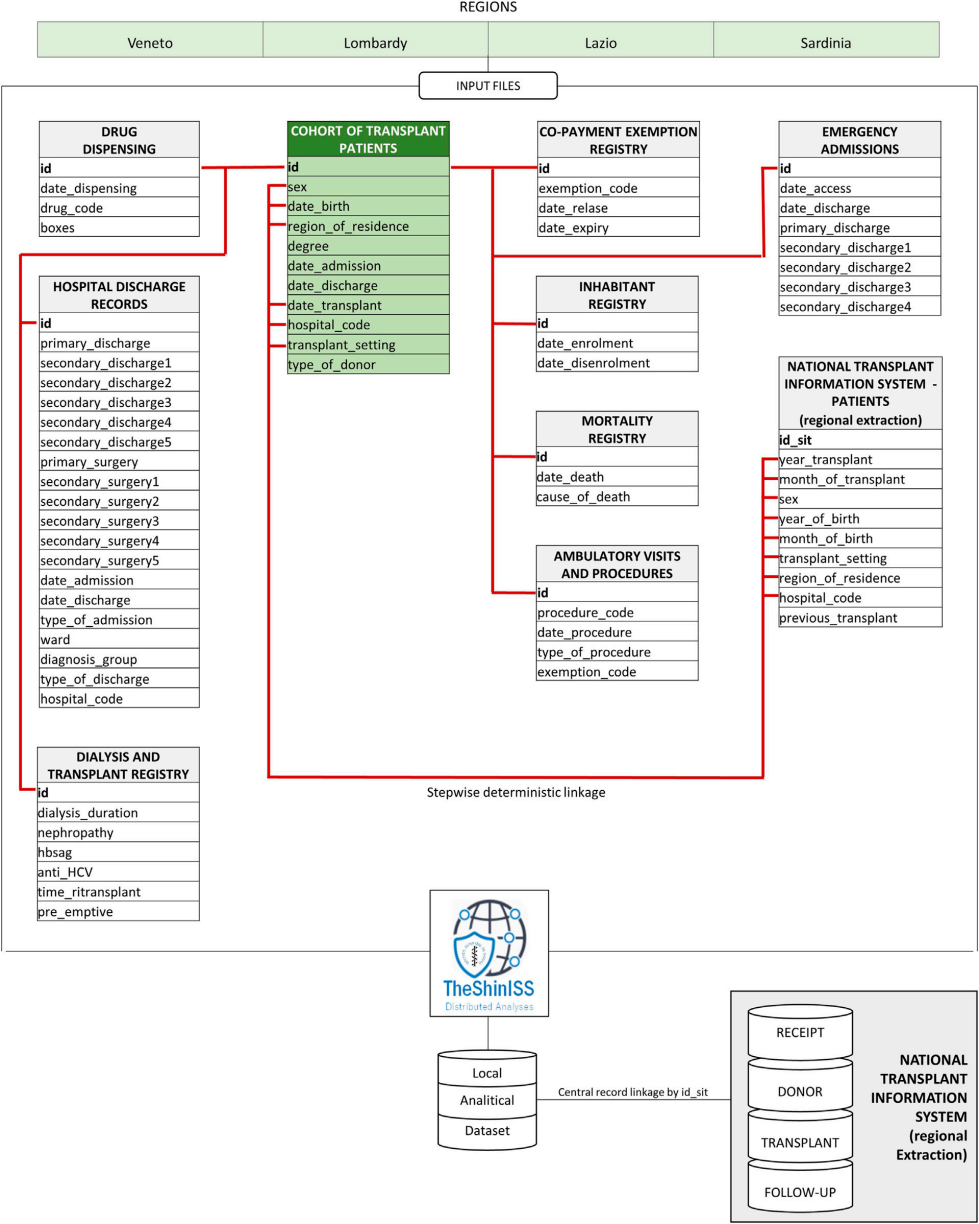
CESIT project distributed data research network using the common data model.

To answer the research objectives, a multi-database cohort study using a Common Data Model (CDM) has been performed (Figure 2). CDM simplifies data management and script development by unifying data into a known form, and applying structural and semantic consistency across multiple datasets. Standardization of structure and content allows the use of centrally developed applications, tools, and methods to combine data to answer a wide range of questions to improve both efficiency and reproducibility.

In particular, regional analytical datasets were created using an open-source tool for distributed analysis that was customized for the purpose of this study.

Specifically, the following administrative data available in the regions involved in this study have been considered:

• Hospital discharge database, containing information on the date of hospital admission and discharge, diagnosis-related group (DRG), principal diagnosis and up to five secondary diagnoses, principal procedure and up to five secondary procedures, coded with International Classification of Diseases, 9th revision, clinical modification (ICD-9-CM).
• Drug dispensing registry, regarding data on out-patient pharmacy database, including data on the date of dispensing, number and cost of dispensed packages, active substance and brand name, coded with Anatomical Therapeutic Chemical (ATC) code and Italian market authorization (AIC) code. Out-of-pocket purchase and inpatient drugs are not be traced.
• Inhabitant registry, including information about the date of birth, gender, date of registration in the regional healthcare system, and, where applicable, date of deregistration.
• Mortality registry, including date and causes of death, coded with international classification of diseases 9th revision-Clinical Modification (ICD-9-CM) codes (where available).
• Emergency department visits database, including reasons for admission to emergency departments, as well as date of admission and discharge.
• Exemptions from healthcare service co-payment database, that contains coded information about chronic diseases or socioeconomic factors.
• Outpatient diagnostic tests and specialist’s visits database, including test or visit specific code, date of test, and name of laboratory where the test is carried out.

All databases can be linked through an anonymous subject identifier.

In addition to administrative databases, regional dialysis and transplant register (RDTR), including information on causes, years, and type of dialysis, are available for two Italian regions (i.e., Lazio and Veneto).

Moreover, clinical information for donor and receiving patients at different timeframes are available at national level through SIT. Specifically, for living and deceased donors and transplant recipients, the available information includes Human Leukocyte Antigen (HLA) type, blood group, Body Mass Index (BMI), and clinical indications to transplant for receiving patients. For the receiving patients, the follow-up includes information on immunosuppressive therapy, organ status (e.g., biopsy, perfusion method), incidence of tumors, blood analyses type, and results (e.g., GFR, BP, proteinuria, and virology).

To link this information system with the transplant cohort, an ad hoc stepwise deterministic record linkage procedure has been defined using pseudonymous information (e.g., sex, organ type, year and month of birth, year and month of transplant, and transplant’s hospital). This procedure is compliant with legislation on data protection and privacy and the principle of data minimization in analytical dataset creation.

The anonymous record linkage approach allows an exact match on a pre-processed subset of personal identifiers. These identifiers are concatenated and encoded into a ‘key,’ which can identify an individual. Subjects with duplicated keys are removed to perform the linkage procedure. Sensitive information fields used in the procedure but not needed for the study are not reported in the analytical dataset.

### Study design and population

**FIGURE 3:**
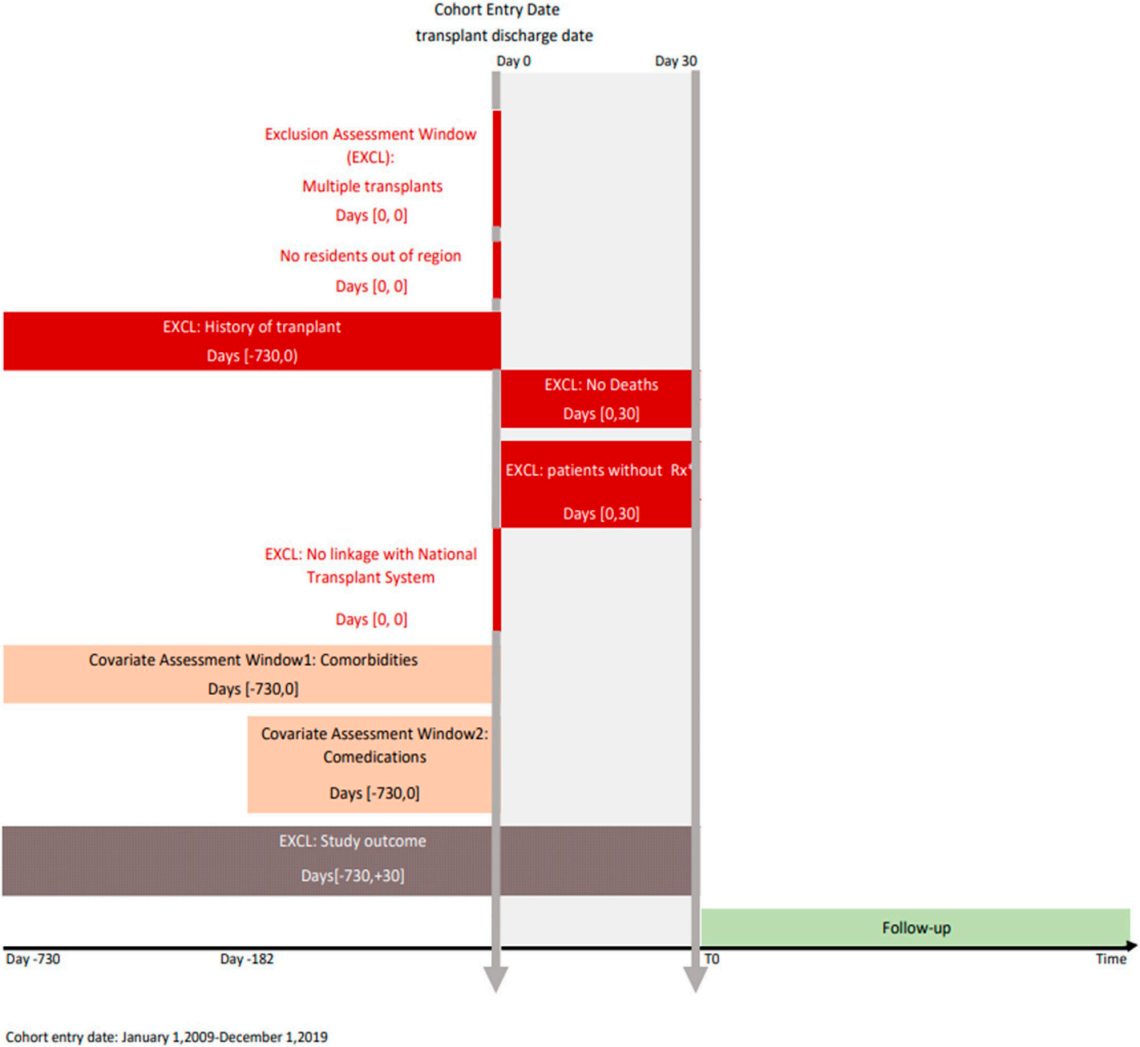
Study design diagram. Note: The study design diagram visually displays study design implementation. The first vertical line represents the cohort entry date (index date) and the second represents the landmark period (30 days). The boxes represent time windows used to perform exclusion or covariate assessment. The brackets in the boxes show time intervals anchored on day 0. Rx, immunosuppressive prescription; EXCL, exclusion.

To date, the study includes all patients who underwent transplant in the period 2009–2019 in the regions considered with at least one immunosuppressive dispensation post-transplant. The graphical depiction of the study design is reported in Figure 3.

Specifically, all transplant patients residing in the catchment areas of all participating regions in the years 2009–2019 have been identified through an algorithm considering all hospitalizations reporting a transplantation procedure (ICD-9-CM codes: kidney 55.6 (excluded 55.61), liver 50.5, heart 37.5, lung 33.5, pancreas 52.8, and intestine 469.7)*.* Only patients with a first hospitalization in the study period have been included. From the resulting cohort, patients with multiple organ transplants and with a transplantation two years previous the discharge date have been excluded. To limit the study population to patients starting immunosuppressive therapy (i.e., incident use), we have considered patients with incident transplantation who have been registered in the regional healthcare system, alive within 30 days after discharge, without any immunosuppressive drug (ATC code: L04) dispensed in the 6 months before transplant discharge date, and who received at least one dispensation of immunosuppressive drugs in the first month after discharge from transplant.

### Maintenance drug treatments

The immunosuppressive maintenance phase has been defined considering the following drugs: CNI: CsA (L04AD01) or TAC (L04AD02); antimet: MMF (L04AA06) or AZA (L04AX01); mTOR: EVE (L04AA18) or SIR (L04AA10); corticosteroids (H02AB07, H02AB04, H02AB06).

Specifically, based on the presence of single or combined dispensations in the window of interest (30 days post-discharge), several regimen groups (TAC or CsA based; with/without steroids) have been identified as index immunosuppressive therapy. For each regimen, the possibility to perform a comparison between generic and brand name drug for CsA, TAC e MMF has been evaluated on the basis of reached frequency. Within the TAC-based regimen, the exposure to prolonged (ER-TAC) and immediate (IR-TAC) formulation has been considered.

Furthermore, to describe the pattern of use over time for each patient, adherence and drug switching has been calculated over several periods of interest (e.g., 6, 12, and 24 months).

### Follow-up

The follow-up period is defined as the period from 30 days post-transplant discharge until the occurrence of one of the following events for each patient (whichever first occurs): death, occurrence of the outcome of interest, transfer out of region, 5 years after the start of follow-up, and end of the study. In the “intention-to-treat” approach, patients will be analyzed according to this definition of follow-up, while in the “as-treated” analysis, patients who interrupt or modify their treatments during follow-up will be censored.

### Outcomes

The following effectiveness outcomes have been considered: mortality, rejection, transplant complications, and organ survival. The selected efficacy outcomes, in particular mortality and rejection, are considered to be robust and are strongly correlated with disease progression. Moreover, other adverse events have been identified, including severe infections, incident diabetes, and tumors.

The time from the start of immunosuppressive drug regimen and following outcome has been assessed by a Kaplan-Meyer curve.

### Patient and donor characteristics

In addition to sex, age, educational level, and the hospital where the transplant has been performed, transplant recipient comorbidities (e.g., hypertension, diabetes, and respiratory and cardiac diseases) and comedications before transplantation (e.g., anticoagulants, antiplatelets, antibiotics, antiarrhythmics, NSAIDs, antiepileptic drugs, antipsychotics) have been recovered retrospectively through administrative databases (Figure 3).

Other clinical variables of interest have been retrieved through the regional dialysis and transplant registries and information transplant system. Specifically, indication for transplant, history of disease, transplant characteristics (e.g., organ characteristics, score, ischemia time, and induction therapy) and demographical (e.g., sex and age), physical (e.g., weight, height, and BMI), clinical characteristics (e.g., haematological/biochemical blood parameters) of donor and recipient have been considered. In particular, the possibility to include in the adjustment models the identified covariates will be evaluated in relation to the type of transplant.

### Analysis

The cohort defined in the previous steps will allow us to:

- Analyze variation among the prescriptive regimens over regions, centers, and years. For each patient, the immunosuppressive therapeutic regimen dispensed after discharge will be defined and the variability per region, per center, and per year will be shown graphically.

- Analyze determinants of the use of different therapeutic regimens. Through multivariate logistic regression models with mixed effects, Median Odds Ratio will be estimated to identify the variability in the choice of immunotherapeutic treatment taking into account the hospital’s role.

- Analyze adherence and switching between therapeutic regimens. Once the post-transplant treatment regimens have been determined, the patients will be followed over time to identify the adherence to different therapeutic regimens [proportion of days covered (PDC) and medication possession ratio (MPR)] and changes in therapy schemes, in terms of possible switches within the same category (CNI, antimet, mTOR inhibitors) or further pharmacological treatments (additive therapeutic schemes).

- Compare risk-benefit profile of different therapies. Different treatment regimens will be compared, taking into account the clinical and demographic characteristics of patients (propensity score/multivariate regression). For each outcome in the study, Hazard Ratios (HR) and their 95% confidence intervals will be calculated by implementing both intention-to-treatment and as-treated analysis.

- Compare use of health resources of different regimes. In particular, the number of hospitalizations for all causes, the number of accesses to the emergency room, and the number of specialist visits will be analyzed.

The analyses will be carried out separately by organ type (i.e., kidney, liver, heart, and lung) and will be repeated by subgroup of population (i.e., pediatric patients and elderly), by drug type (i.e., generic or originator), and by formulation (i.e., immediate or prolonged release).

Moreover, the availability of SIT data offers the opportunity to estimate the level of concordance between immunosuppressive pattern reported by physician, available in national transplant information system, and those obtained by pharmaceutical claims.

## Results

### Flow chart

**FIGURE 4:**
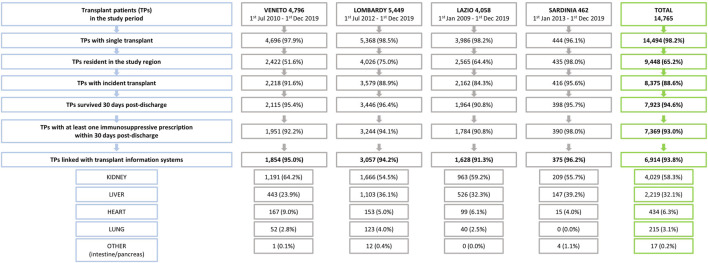
Study design including inclusion/exclusion criteria by region and overall.

The flow chart in Figure 4 shows the selection process of the study cohort. Of the 14,765 patients discharged from hospital with an organ solid transplant procedure between 1 January 2007 and 1 December 2019, 7,369 (49.9%) met the inclusion criteria. Among those, it was possible to link data for 93.8% with SIT using a stepwise deterministic record linkage procedure.

### Characteristics

Overall, 6,914 transplant patients from 2009–19 were identified: 4,029 (58,3%) kidney patients, 2,219 (32.1%) liver patients, 434 (6.3%) heart patients, and 215 (3.1%) lung patients (Table 1). The percentage of males were 61.6%, 76.8%, 64.6%, and 66.5%, respectively. Mean age was 52.1 years for kidney patients, 52.7 years for liver patients, 43.7 years for heart patients, and 44.7 years for lung transplant patients; in the heart cohort a higher contribution of pediatric patients was observed (15.9% with age<18 years). Transplant hospital stay length mean ranged from 17.3 days for kidney to 44.1 days for heart recipients. For donors, the percentage of living patients was 10.8% in kidney and less of 1% in liver transplant, and the main causes of death was cerebral hemorrhage for all types of organ transplants. The demographical donor characteristics were very different among types of organ transplants: the percentage of female was lower for kidney transplant (10.4% compared to 44.0%, 36.4%, and 43.3% for liver, heart, and lung, respectively), while donor age was higher in kidney and liver donors (55.8 and 57.9 years compared to 36.1 and 40.8 years for heart and lung, respectively), the percentage of pediatric donors in heart and lung cohorts was 16.1% and 11.2%, respectively.

**TABLE 1:** Demographic and clinical characteristics of donor and recipient by type of organ transplant.

	Kidney		Liver		Heart		Lung	
	4,029		2,219		434		215	
	N	%	N	%	N	%	N	%
Sex (recipient)								
M	2,599	64.5%	1717	77.4%	306	70.5%	143	66.5%
F	1,430	35.5%	502	22.6%	128	29.5%	72	33.5%
Age (recipient)								
<18 years	110	2.7%	107	4.8%	69	15.9%	10	4.7%
18–64 years	3,112	77.2%	1828	82.4%	336	77.4%	194	90.2%
65 + years	807	20.0%	284	12.8%	29	6.7%	11	5.1%
Mean (SD) years	52.1 (14.3)	52.7 (14.0)	43.7 (19.1)	44.7 (15.6)				
BMI (recipient)								
Underweight	264	6.6%	124	5.7%	67	15.7%	33	18.2%
Normal	2,155	53.5%	923	42.4%	229	53.5%	91	50.3%
Overweight	1,250	31.0%	824	37.9%	103	24.1%	43	23.8%
Obese	357	8.9%	305	14.0%	29	6.8%	14	7.7%
Missing	3		43		6		34	
Transplant hospital stay length								
Mean (SD) days	17.3 (10.3)	24.7 (20.1)	44.1 (31.9)	35.9 (21.9)				
Donor								
Living	421	10.4%	18^∗^	0.8%	0	0.0%	0	0.0%
Dead	3,608	89.6%	2,201	99.2%	434	100.0%	215	100.0%
Cause of death (donor)								
Cerebral hemorrhage	1997	55.3%	1,254	57.0%	165	38.0%	103	47.9%
Post-anoxic encephalopathy	416	11.5%	237	10.8%	45	10.4%	14	6.5%
Ischemic stroke	307	8.5%	181	8.2%	16	3.7%	11	5.1%
Other	888	24.6%	529	24.0%	208	47.9%	87	40.5%
Sex (donor)								
M	2,157	53.5%	1,243	56,0%	276	63.6%	122	56.7%
F	1,872	46.5%	976	44,0%	158	36.4%	93	43.3%
Age (donor)								
<18 years	128	3.2%	79	3.6%	70	16.1%	24	11.2%
18–64 years	2,490	61.8%	1,169	52.7%	351	80.9%	183	85.1%
65 + years	1,411	35.0%	971	43.8%	13	3.0%	8	3.7%
Mean (SD) years	55.8 (17.1)	57.9 (18.7)	36.1 (17.3)	40.8 (15.8)				
BMI (donor)								
Underweight	108	2.7%	62	2.8%	37	8.5%	9	4.2%
Normal	1806	45.0%	943	42.9%	211	48.6%	139	64.7%
Overweight	1,516	37.8%	890	40.5%	143	32.9%	49	22.8%
Obese	584	14.5%	301	13.7%	43	9.9%	18	8.4%
Missing	15		23		0		0	
Infections (donor)^∗^	616	17.1%	291	14.9%	50	11.5%	14	6.5%
Neoplasm/neoplasia (donor)^∗^	126	3.5%	114	5.2%	8	1.9%	7	3.3%
Indications for transplant								
Glomerular nephropathies	1719	42.7%						
Cystic nephropathies	803	19.9%						
Hypertensive nephrosclerosis	347	8.6%						
Tubular and interstitial nephropathies	273	6.8%						
Diabetic nephropathy	222	5.5%						
Cirrhosis			1,251	56.4%				
Malignant neoplasms			707	31.9%				
Cardiomyopathies					257	59.2%		
Coronary artery disease					110	25.3%		
Cardiopathies					41	9.4%		
Cystic fibrosis							80	37.2%
Idiopathic pulmonary fibrosis							53	24.7%
Emphysema/COPD°							32	14.9%
Other	665	16.5%	261	11.8%	26	6.0%	50	23.3%
Charlson index								
0–1	3,280	81.4%	762	34.3%	226	52.1%	186	86.5%
2	612	15.2%	1,048	47.2%	136	31.3%	24	11.2%
3+	137	3.4%	409	18.4%	72	16.6%	5	2.3%
Comorbidities and comedications								
Cancer	258	6.4%	72	3.2%	24	5.5%	20	9.3%
Diabetes	533	13.2%	642	28.9%	57	13.1%	55	25.6%
Thyroid disease	521	12.9%	119	5.4%	83	19.1%	8	3.7%
Lipid metabolism disorders	235	5.8%	82	3.7%	43	9.9%	11	5.1%
Hypertension	2,860	71.0%	440	19.8%	109	25.1%	35	16.3%
Depression	201	5.0%	125	5.6%	36	8.3%	29	13.5%
Infections	163	4.0%	162	7.3%	18	4.1%	9	4.2%
Anticoagulants	434	10.8%	275	12.4%	206	47.5%	31	14.4%
Antiplatelet	1,354	33.6%	92	4.1%	155	35.7%	24	11.2%
Statins	1,581	39.2%	259	11.7%	130	30.0%	23	10.7%

The main indication for transplant were glomerular (42.7%) and cystic nephropathies (19.9%) for kidney recipients, cirrhosis (56.4%) and hepatocarcinoma (31.9%) for liver recipients (deep variations over time have been observed after the introduction of treatment with direct-acting antiviral agents for HCV in 2016), cardiomyopathy (59.2%) and coronary artery disease (25.3%) for heart recipients, and cystic (37.2%) and idiopathic pulmonary fibrosis (24.7%) for lung recipients.

Overall, a higher comorbidity index was detected for liver and heart recipients. Specifically, diabetes was more frequent in liver and lung transplant patients (28.9% and 25.6%, respectively), while hypertension was higher in kidney recipients (71.0%).

### Index treatment

**FIGURE 5:**
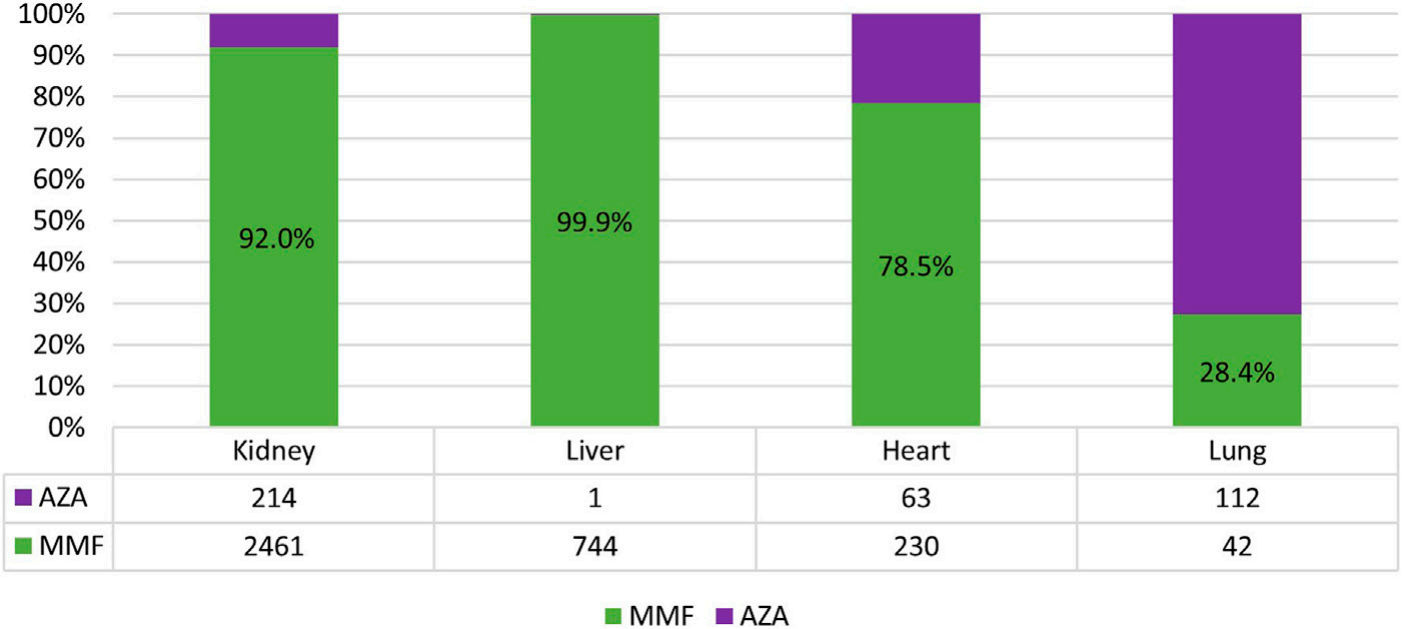
Percentage of mycophenolate (MMF) or azathioprine (AZA) use among antimetabolite users.

As shown in Table 2, in kidney, liver, and lung organ transplant settings, most patients received TAC-based therapy (kidney-Tx: 70.4%; liver-Tx: 84.1%; lung-Tx: 57.2%), usually in association with corticosteroids (more than 80% of cases). Specifically, among TAC users, the most dispensed therapy was TAC + antimet + prednisone for kidney (1,682/2,835 = 59.3%) and lung recipients (78/123 = 63.4%), and TAC + prednisone (786/1867 = 42.1%) for liver recipients. The antimet mainly used was MMF in kidney (92.0%), liver (99.9%), heart (78.5%), and AZA in lung recipients (72.6%) (Figure 5).

**TABLE 2:** Immunosuppressive maintenance therapy post-discharge by type of organ transplant.

	Kidney			Liver			Heart			Lung		
	N	%	+cortico steroids	N	%	+cortico steroids	N	%	+cortico steroids	N	%	+cortico steroids
TAC-based	2,835	70.4	2,315	1867	84.1	1,521	66	15.0	59	123	57.2	114
Mono	261	6.5	210	927	41.8	786	21	4.8	19	42	19.5	36
+ antimet	1980	49.1	1,682	690	31.1	579	45	10.3	40	81	37.7	78
+ mTOR	559	13.9	398	211	9.5	129	—	—	—	—	—	—
Other	35	0.9	25	39	1.8	27	—	—	—	—	—	—
CsA-based	787	19.5	564	271	12.2	198	333	77.0	258	76	35.3	71
Mono	85	2.1	74	225	10.1	162	84	19.8	64	13	6.0	12
+ antimet	617	15.3	416	39	1.8	34	231	52.8	181	63	29.3	59
+ mTOR	79	2.0	69	7	0.3	2	14	3.4	10	—	—	—
Other	6	0.1	5	—	—	—	4	0.9	3	—	—	—
No-CNI	407	10.1	381	81	3.7	69	35	8.0	26	16	7.4	6
+ antimet	78	1.9	60	16	0.7	12	17	3.9	9	10	4.7	6
+ mTOR	30	0.7	27	16	0.7	11	2	0.5	1	—	—	—
Other	299	7.4	294	49	2.2082	46	16	3.7	16	6	2.8	6
Total	4,029	100%	3,260	2,219	100%	1788	433	100%	343	215	100%	191

**FIGURE 6:**
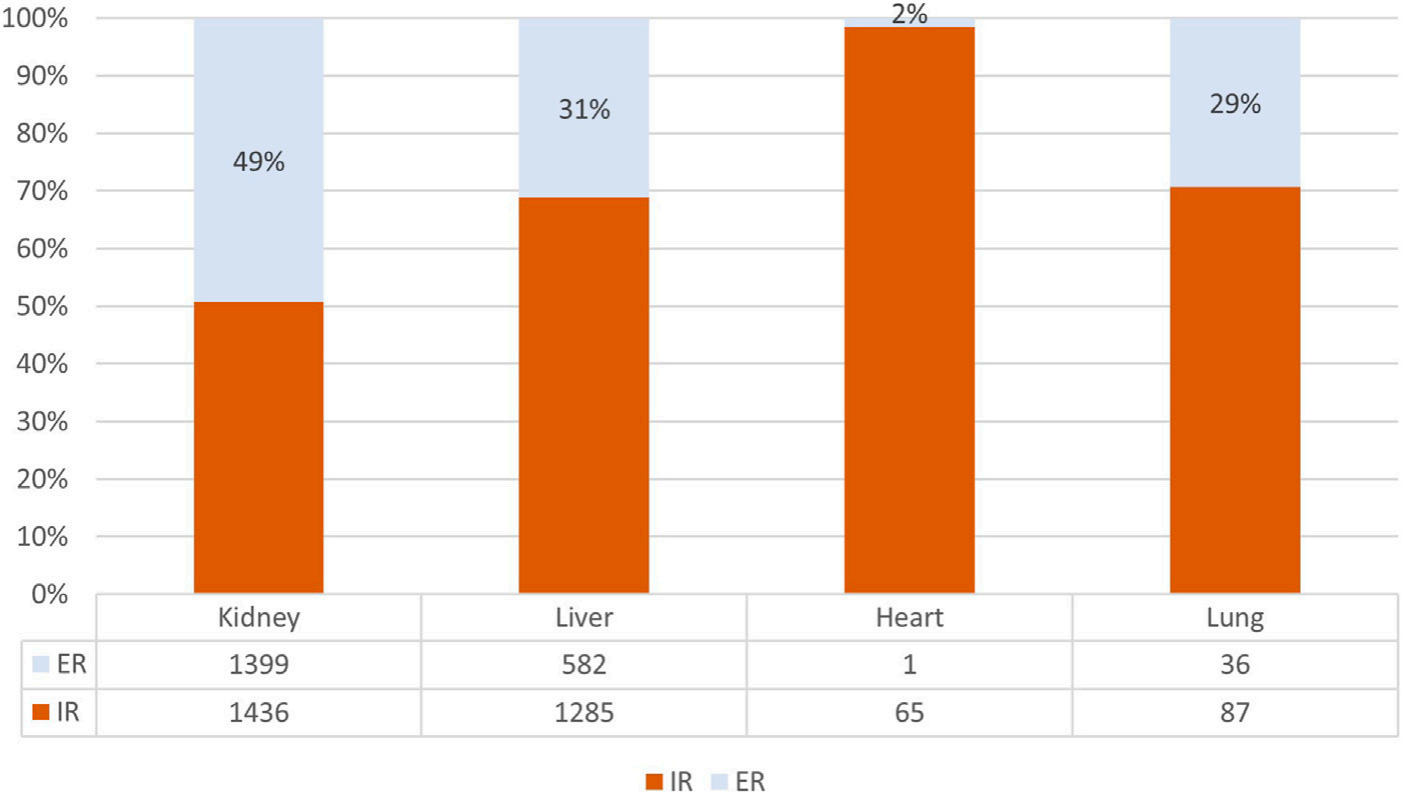
Percentage of immediate (IR) or extended release (ER) use among tacrolimus users.

In kidney and liver settings, the use of TAC + mTOR combination was reserved to 13.9% and 9.5% of cases (Table 2), respectively, and for both organ types the most dispensed TAC formulation post-transplant discharge was immediate release formulation (kidney-Tx: 50.7%; liver-Tx:68.8%) (Figure 6).

Heart recipients were treated mainly with CsA-based therapy (77.0%), among those the most frequent drug combination was CsA + antimet + prednisone (181/333 = 54.4%).

The percentage of kidney and liver patients treated with TAC-based therapy increased over time (kidney-Tx: 2009: 57.0% 2019: 82.2%; liver-Tx: 2009: 84.4% 2019: 92.9%). The percentage of heart transplant patients treated with CsA has decreased in the last few years (Supplementary Table S1).

For all types of organ transplants, a small but interesting percentage of patients with immunosuppressive therapy without CNI was observed (kidney-Tx: 10.1%; liver-Tx:3.7%; heart-Tx: 8.0%; lung-Tx:7.4%)—mainly steroid-based.

**FIGURE 7:**
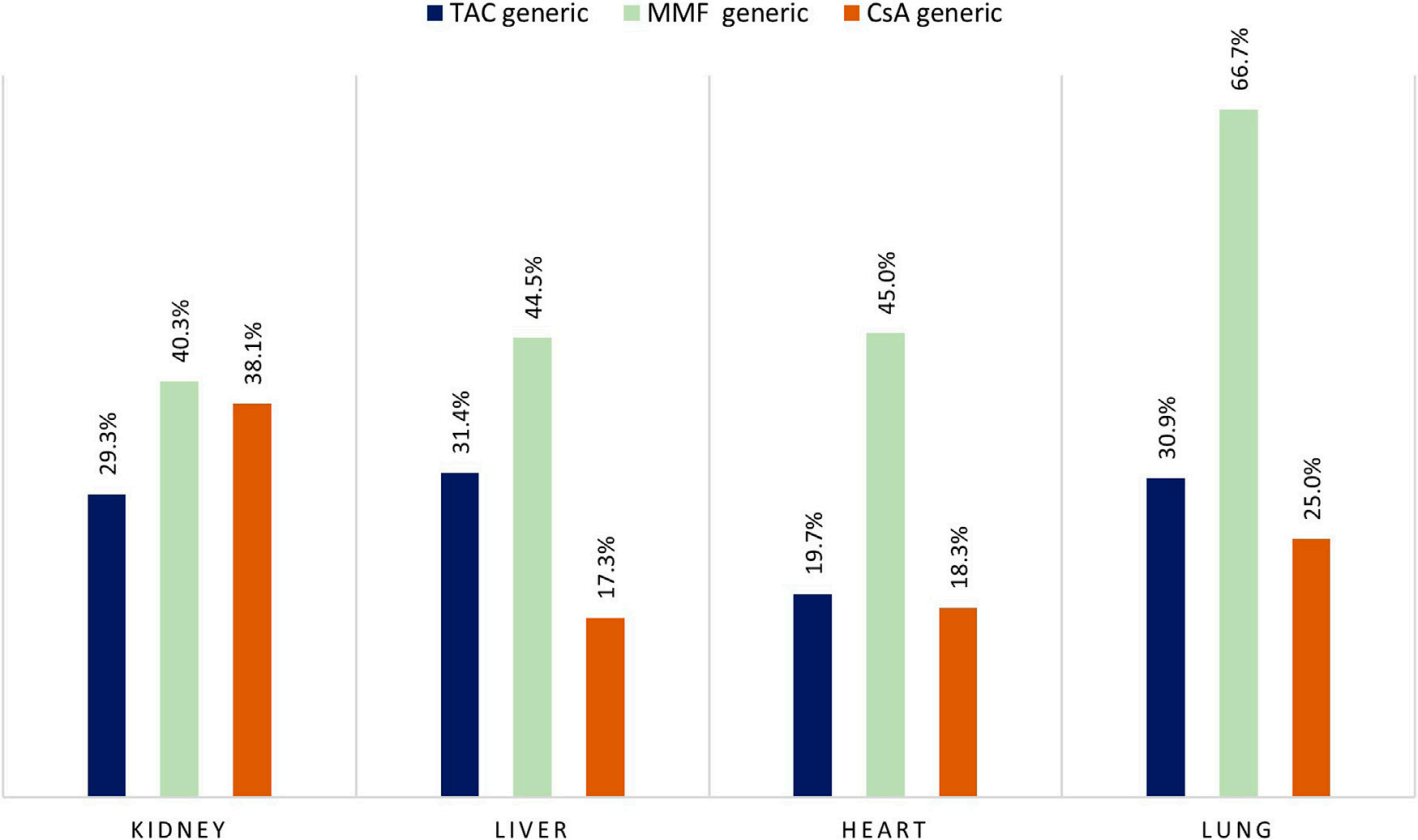
Percentage of branded versus generic drug for tacrolimus (TAC), mycophenolate (MMF), and cyclosporine (CsA) by type of organ transplant. Note: Only patients enrolled in the period where both brand and generic version of the drug were available were included in the analysis.

During the study period, the adoption of generic immunosuppressant medication varied for organ type and active agent (Figure 7). Specifically, the percentage of patients who were dispensed generic version of TAC was highest in liver recipients (31.4%) and lowest in heart recipients (19.7%), while for MMF we observed a generic uptake of more 40% for all type of organ transplants with a peak in use for the lung recipients (66.7%).

### Observation period and outcomes

**FIGURE8:**
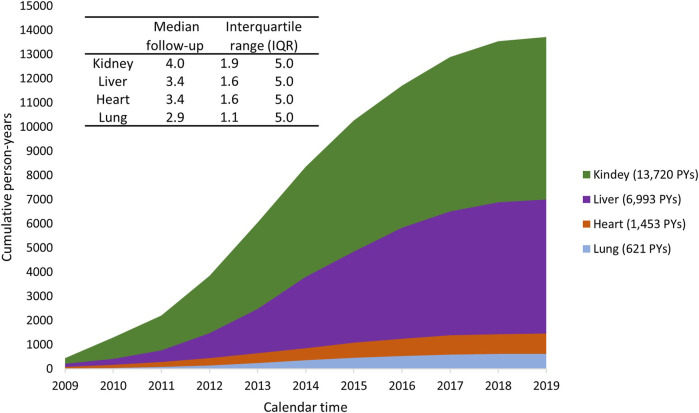
Cumulated person-years (PYs) during the study period 2009–2019, stratified by type of organ transplant.

Person years during the observation period increased gradually for all settings (Figure 8). Specifically, to date, each individual has been followed for a maximum of 5 years. The median follow-up period was 4.0 years for kidney recipients, 3.4 years for liver recipients, 3.4 years for heart recipients, and 2.9 years for lung recipients.

**FIGURE 9:**
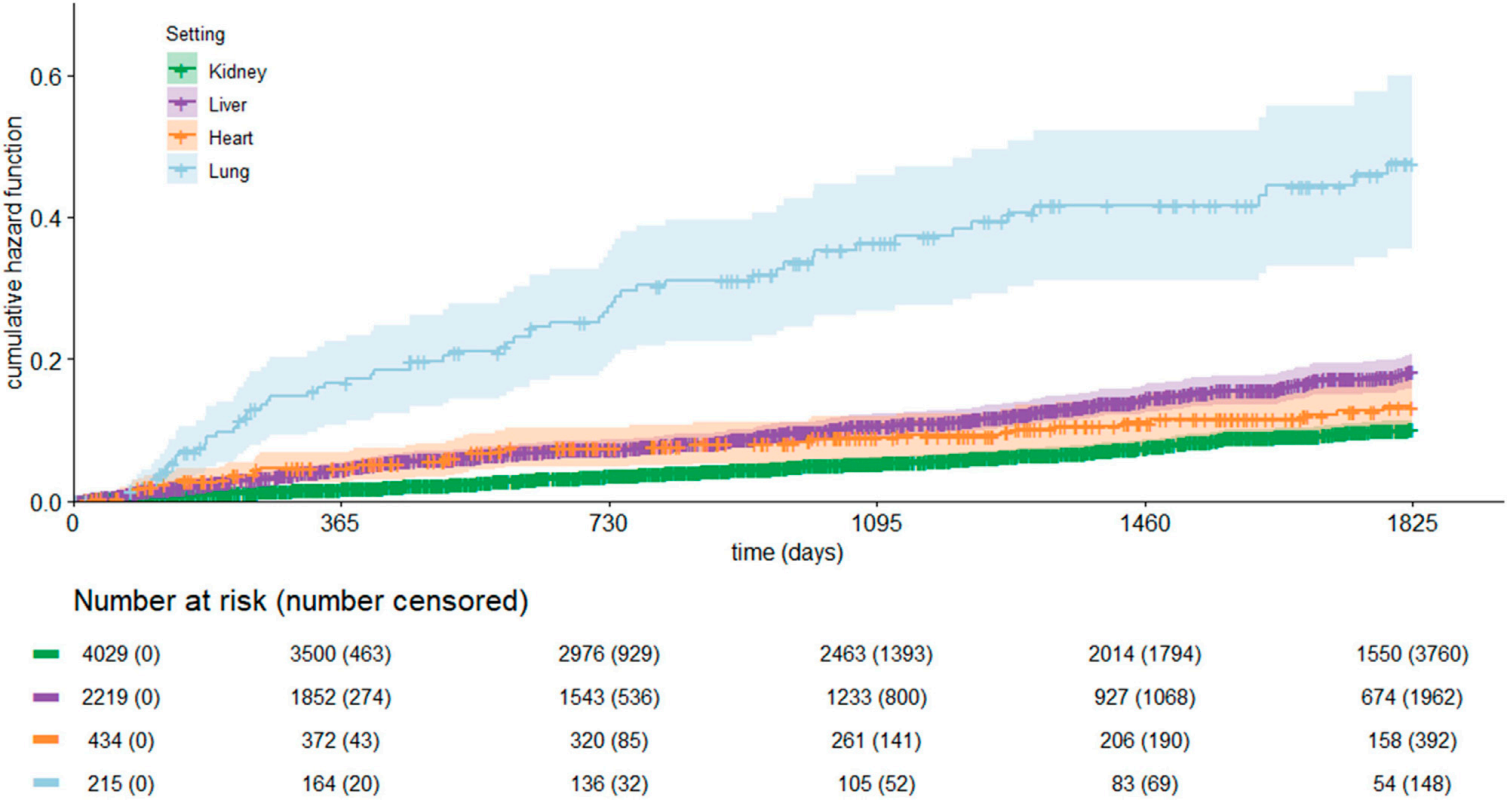
Kaplan-Meier curve for all-cause mortality by type of organ transplant.

The Kaplan–Meier curve for all-cause mortality by setting is shown in Figure 9. The cumulative incidence rate was higher in lung recipients, followed by liver, heart, and kidney recipients. During follow-up, mortality occurred in 269 patients in kidney recipients (Incidence Rate, IR = 1.96/100 PYs), 42 patients in heart recipients (IR = 2.89/100 PYs), 257 patients in liver recipients (IR = 3.67/100 PYs), and 67 in lung recipients (IR = 10.79/100 PYs) (Table 3). Higher incidence rates of reject/graft failure, infection, and diabetes were observed in lung transplants, while the incidence of cancer was higher for liver recipients (IR = 5.95/100 PYs).

**TABLE 3:** Incidence rate by transplant setting.

	N	Incident cases (n)	Person years (PYs)	Incidence per 100 PYs	CI95%		N	N	Incident cases (n)	Incidence per 100 PYs	CI95%	
	Kidney						Liver					
Mortality	4,029	269	13,720.5	1.96	1.90	2.02	2,219	257	6,993.2	3.67	3.52	3.83
Reject/graft failure	3,471	254	11,987.9	2.12	2.05	2.19	2,108	105	5,984.2	1.75	1.68	1.83
Infections	3,429	898	9,571.4	9.38	9.07	9.70	1846	225	5,307.1	4.24	4.05	4.43
Diabetes	3,280	373	10,303.2	3.62	3.50	3.74	1,327	156	3,714.1	4.20	3.97	4.43
Cancer	3,764	295	12,475.1	2.36	2.29	2.44	2,123	362	6,085.7	5.95	5.70	6.20
	**Heart**						**Lung**					
Mortality	434	42	1,452.8	2.89	2.62	3.16	215	67	620.8	10.79	9.35	12.24
Reject/graft failure	428	18	1,330.9	1.35	1.22	1.48	213	39	585.7	6.66	5.76	7.55
Infections	338	77	968.2	7.95	7.11	8.80	120	68	181.9	37.38	30.69	44.07
Diabetes	359	25	1,156.9	2.16	1.94	2.38	120	43	248.3	17.32	14.22	20.42
Cancer	409	21	1,329.7	1.58	1.43	1.73	194	17	547.8	3.10	2.67	3.54

## Discussion

The CESIT network provides a unique opportunity to study several aspects related to the use, safety, and effectiveness of post-transplant maintenance immunosuppressive drug therapy in real practice. Considering the data that have been made available to date, we have information on about 7.000 transplant patients. In this context, we observed a wide variability in immunosuppressive therapy among organs: TAC-based therapies were more frequently prescribed in kidney, liver, and lung recipients, although combinations with other immunosuppressive drugs were different in the three groups, while CsA-based therapies were more frequently prescribed in heart transplants. Our preliminary results are in line with the international literature, which identifies triple therapy, in terms of TAC + MMF + corticosteroids, as the most prescribed in the renal setting (Axelrod et al., 2016; Hart et al., 2016; Chang et al., 2017), and CNI-based two-drug regimen, in terms of TAC + MMF, as the favorable maintenance immunosuppression in liver transplantation (Huang et al., 2016). In patients with heart transplant, CsA remained the most frequently used calcineurin inhibitors but a slight decrease over time was detected. This phenomenon is in agreement with a previous study where a gradual increase in TAC has been shown (Chou et al., 2014). In the lung setting, we detected an immunosuppressive off label use, in terms of TAC and/or MMF, common in clinical practice (De Cos et al., 2015).

Moreover, the focus on the use of different TAC formulations and the adoption of generic versions of immunosuppressive drugs showed variability across organ transplant settings that requires further investigation. This might help audit process and discussions useful to improve appropriate use of resources.

Furthermore, the need for research networks in the transplant context that are able to study specific issues (e.g., drug appropriateness, adherence, and switching) and compare the risk-benefit profile of drugs in real-world practice is increasingly recognized (Massie et al., 2014; Lorent et al., 2019; Sahman et al., 2021).

The U.S. Food and Drug Administration has recently approved new use for Prograf (tacrolimus) in lung transplantation based on an observational study that provided real-world evidence of its effectiveness to prevent organ rejection (Erdman et al., 2021). This approval reflects how well-designed, non-interventional studies can play a significant role in regulatory decision-making, supporting new drug indication, confirming evidence of effectiveness, and detecting potential adverse effects in clinical practice.

In Italy, during the last couple of years, many research networks have been created to study pharmacoepidemiologic and pharmacovigilance issues in several clinical areas, such as the monitoring of medication use during pregnancy (Belleudi et al., 2021), the post-marketing surveillance of biological drugs (Trifirò et al., 2021), and the assessment of the association between drugs, vaccines, and COVID-19 (Ferroni et al., 2020; Trifirò et al., 2020; Massari et al., 2021; Spila Alegiani et al., 2021).

The strength of these networks is guaranteed by the availability of a large amount of electronic data in terms of administrative and clinical databases, the use of specific tools for distributed analyses through a common data model framework, the involvement of multidisciplinary team, and the opportunity to rapidly update research protocols to accumulate evidence on emerging issues, such as COVID-19 research.

In the transplant context, the current COVID-19 pandemic has given rise to many questions about the management of immunosuppressive medication in transplant patients. In fact, solid organ transplant recipients may be at increased risk for COVID-19 because they are immunosuppressed and are less likely to mount effective immune responses to vaccination. To understand the role of immunosuppressive therapy in COVID-19, infection risk and vaccine effectiveness are key elements for the management of immunosuppressive therapy in transplant patients during the pandemic, including those with moderate to severe COVID-19. The CESIT network will give us the opportunity, once the data have been updated, to implement specific analyses regarding the direct and indirect impact of COVID-19 in this population, as well as effectiveness and safety of COVID-19 vaccines.

### Strengths and limitations

This study’s limitations include those inherent to the observational studies based on claims data, such as the inability to track unreimbursed over-the-counter dispensations, the presence of unmeasured confounders in the comparative-effectiveness analysis, and the difficulty of detecting the actual dosage prescribed by physician to patients. In particular, to investigate the accuracy of drug regimen retrieved from dispensation data, an analysis of information concordance has been planned to compare these patterns with treatment data available on SIT. Furthermore, the presence of multidisciplinary team, including experts in biostatistics and pharmacoepidemiology, ensures us that the most sophisticated statistical methods will be applied to minimize bias and provide more reliable findings from which conclusions can be drawn and decisions made.

## Conclusion

The CESIT research network represents a great opportunity to have an integrated data system on donor and recipient demographic and clinical characteristics, transplantation, maintenance immunosuppressive therapy, and outcomes. The longitudinal nature of data collected within the network will allow us to monitor post-transplant immunosuppressive therapy, to show intra and inter-regional variability in dispensation patterns, and to compare the risk-befit profile of different therapeutic strategies.

## CESIT study group

Antonio Addis, Nera Agabiti, Stefania Spila Alegiani; Valeria Belleudi; Massimo Cardillo; Paolo Carta, Marina Davoli, Michele Ercolanoni, Eliana Ferroni, Pamela Fiaschetti, Marco Finocchietti, Bedeschi Gaia, Donatella Garau; Valentina Ientile, Ursula Kirchmayer, Luca L’Abbate; Stefano Ledda, Lorella Leoni; Olivia Lombardozzi; Ersilia Lucenteforte, Claudia Lucia Marino, Maria Marino, Lucia Masiero, Marco Massari, Arianna Mazzone, Ugo Moretti; Andrea Angelo Nisic, Maurizio Nordio, Alessandra Oliveti, Daniela Peritore, Giuseppe Piccolo, Silvia Pierobon, Francesca R Poggi, Francesca Puoti, Andrea Rosa, Alessandro C. Ricci, Vito Sparacino, Matilde Tanaglia, Silvia Trapani, Gianluca Trifirò, Martina Zanforlini, Manuel Zorzi.

## Data Availability

The datasets generated and/or analyzed during the current study are not publicly available because of privacy reasons.
