# Supplementary material for: An Italian multicentre distributed data research network to study the use, effectiveness, and safety of immunosuppressive drugs in transplant patients: Framework and perspectives of the CESIT project
Source: Front Pharmacol. 2022 Sep 15;13:959267. doi: 10.3389/fphar.2022.959267 (PMC9521186; doi:10.3389/fphar.2022.959267)
Supplement: Supplementary file 1 [file Table1.docx]

Table S1. Percentage of tacrolimus-, cyclosporine- and no calcineurin inhibitor- based therapy by year of discharge and setting

| **Year of discharge** | **2009** | **2010** | **2011** | **2012** | **2013** | **2014** | **2015** | **2016** | **2017** | **2018** | **2019** |
| --- | --- | --- | --- | --- | --- | --- | --- | --- | --- | --- | --- |
| Kidney (N) | 93 | 178 | 189 | 348 | 457 | 475 | 454 | 436 | 501 | 476 | 422 |
| TAC-based | 57.0% | 52.2% | 72.5% | 57.5% | 63.0% | 65.9% | 72.9% | 70.6% | 76.4% | 80.3% | 82.2% |
| CsA-based | 25.8% | 21.3% | 12.2% | 22.4% | 25.2% | 22.9% | 19.2% | 19.3% | 18.0% | 17.2% | 13.5% |
| No CNI | 17.2% | 26.4% | 15.3% | 20.1% | 11.8% | 11.2% | 7.9% | 10.1% | 5.6% | 2.5% | 4.3% |
|  |  |  |  |  |  |  |  |  |  |  |  |
| Liver (N) | 45 | 45 | 81 | 159 | 227 | 288 | 256 | 302 | 287 | 276 | 253 |
| TAC-based | 84.4% | 82.2% | 86.4% | 71.7% | 72.7% | 75.3% | 77.3% | 90.4% | 94.1% | 90.6% | 92.9% |
| CsA-based | 2.2% | 4.4% | 9.9% | 22.6% | 22.5% | 20.5% | 19.5% | 8.3% | 4.9% | 5.8% | 3.6% |
| No CNI | 13.3% | 13.3% | 3.7% | 5.7% | 4.8% | 4.2% | 3.1% | 1.3% | 1.0% | 3.6% | 3.6% |
|  |  |  |  |  |  |  |  |  |  |  |  |
| Heart (N) | 18 | 18 | 26 | 35 | 46 | 47 | 55 | 52 | 58 | 38 | 41 |
| TAC-based | 11.1% | 5.6% | 19.2% | 5.7% | 13.0% | 6.4% | 16.4% | 11.5% | 13.8% | 42.1% | 19.5% |
| CsA-based | 77.8% | 83.3% | 61.5% | 91.4% | 80.4% | 89.4% | 80.0% | 86.5% | 77.6% | 47.4% | 61.0% |
| No CNI | 11.1% | 11.1% | 19.2% | 2.9% | 6.5% | 4.3% | 3.6% | 1.9% | 8.6% | 10.5% | 19.5% |
|  |  |  |  |  |  |  |  |  |  |  |  |
| Lung (N) | 4 | 4 | 12 | 15 | 32 | 25 | 30 | 27 | 27 | 19 | 20 |
| TAC-based | 25.0% | 0.0% | 16.7% | 26.7% | 62.5% | 84.0% | 70.0% | 40.7% | 70.4% | 57.9% | 65.0% |
| CsA-based | 75.0% | 100.0% | 83.3% | 66.7% | 34.4% | 12.0% | 26.7% | 37.0% | 18.5% | 36.8% | 25.0% |
| No CNI | 0.0% | 0.0% | 0.0% | 6.7% | 3.1% | 4.0% | 3.3% | 22.2% | 11.1% | 5.3% | 10.0% |
